# Seasonal Variations of Community Structure and Functional Genes of *Synechococcus* in the Subtropical Coastal Waters: Insights from FACS and High-Throughput Sequencing

**DOI:** 10.3390/microorganisms13040764

**Published:** 2025-03-27

**Authors:** Zhenzhen Song, Ting Zhang, Yantao Liang, Andrew Mcminn, Min Wang, Nianzhi Jiao, Tingwei Luo

**Affiliations:** 1 College of Marine Life Sciences, Ocean University of China, Qingdao 266003, China; songzhenzhen@stu.ouc.edu.cn (Z.S.); andrew.mcminn@utas.edu.au (A.M.); mingwang@ouc.edu.cn (M.W.); 2 Fujian Key Laboratory of Marine Carbon Sequestration, Carbon Neutral Innovation Research Center, Xiamen University, Xiamen 361102, China; 22320181152153@stu.xmu.edu.cn (T.Z.); jiao@xmu.edu.cn (N.J.); 3 Institute of Evolution and Marine Biodiversity, MoE Key Laboratory of Evolution and Marine Biodiversity, Frontiers Science Center for Deep Ocean Multispheres and Earth System, Center for Ocean Carbon Neutrality, Ocean University of China, Qingdao 266003, China; 4 UMT-OUC Joint Centre for Marine Studies, Qingdao 266003, China; 5 Institute for Marine and Antarctic Studies, University of Tasmania, Hobart 7001, Tasmania, Australia; 6 Haide College, Ocean University of China, Qingdao 266100, China

**Keywords:** fluorescence-activated cell sorting, high-throughput sequencing, *Synechococcus* community structure, functional genes, seasonal variations

## Abstract

*Synechococcus* plays a pivotal role in the marine biogeochemical cycle. Advances in isolation techniques and high-throughput sequencing have expanded our understanding of the diversity of the *Synechococcus* community. However, their genomic diversity, functional dynamics and seasonal variations in the coastal waters are still not well known. Here, seawater samples were collected seasonally (March, June, August, December) from three stations in the coastal waters of Xiamen. Using fluorescence-activated cell sorting (FACS), we isolated 1000 *Synechococcus* cells per sample and performed ITS amplicon sequencing and metagenomic sequencing to analyze the seasonal variations in community structure and functional genes of *Synechococcus*. Firstly, we conducted a comparative analysis of in situ data and FACS data from three sampling sites in August. FACS samples revealed low-abundance *Synechococcus* strains underdetected by in situ samples. In addition, 24 clades representing *Synechococcus* subclusters S5.1, S5.2, and S5.3 were detected from three in situ samples and twelve FACS samples, suggesting the high diversity of *Synechococcus* in the coastal waters of Xiamen. Furthermore, the *Synechococcus* community displayed pronounced seasonal variations, and temperature significantly influenced the variations in *Synechococcus* community composition. Additionally, *Synechococcus* populations exhibit seasonal functional dynamics, with enhanced metabolic activity in summer characterized by higher numbers of functional genes associated with metabolic pathways compared to winter samples. Altogether, this study underscored the significance of FACS and high-throughput sequencing to reveal the diversity and functional dynamics of *Synechococcus*.

## 1. Introduction

The *Synechococcus* genus of cyanobacteria, a key photosynthetic autotroph, is ubiquitous on Earth. It significantly contributes to approximately 20% of primary productivity in oceans, and *Synechococcus* interactions with microbial heterotrophs partly underlie the marine microbial food loop [1,2]. Dissolved organic matter (DOM) released from *Synechococcus* directly links the carbon fixed by primary producers to the marine DOM pool, playing a vital role in marine carbon cycles and biogeochemical processes [3]. *Synechococcus* is particularly abundant in marine environments, with concentrations reaching up to 10^6^ cells/mL in coastal and up-welling areas [4,5]. Phylogenetic analysis of the 16S rRNA gene reveals three major subclusters within *Synechococcus*: S5.1, S5.2, and S5.3, showcasing rich diversity. Further characterization of *Synechococcus* phylogenetic diversity can be achieved using the16S-23S internal transcribed spacer (ITS) region and marker genes such as *cpeA*, *narB*, *ntcA*, *petB*, *rbcL*, and *rpoC1* [6,7]. Existing studies have confirmed the identification of over twenty clades across the three subclusters [8,9]. The phylogenetic diversity of *Synechococcus* may represent physiological or ecological diversity. For instance, strains from clade III exhibit unique motility characteristics, while clades CRD2 and XV may have limited growth on nitrate or reduced growth rates [10,11]. Although different clades display distinct ecological diversity, the coexistence of various *Synechococcus* clades is commonly observed in certain marine regions [12,13].

Alterations in the marine environment, such as temperature, nutrient availability, light conditions and so on, are bound to result in the change and adaptation of biological populations. Microbial community structures in a given study area are often influenced by environmental factors [14,15], and *Synechococcus* populations are no exception, exhibiting dynamic variations in both temporal and spatial scales [16]. *Synechococcus* exhibits remarkable adaptability to environmental factors, particularly in adapting to temperature changes, so the abundance and proportion in the phytoplankton community may rise even further [17,18]. Environmental fluctuations drive dynamic modifications in physiological and gene regulation of *Synechococcus* and may further lead to its adaptive evolution [19].

The common approach for studying microorganisms is laboratory cultivation. However, the cultivable fraction of microorganisms, particularly in marine environments, is limited due to the constraints of cultivation conditions [20]. As a result, the information obtained from laboratory-cultivated *Synechococcus* is limited. Advancements in high-throughput sequencing have enabled the acquisition of more *Synechococcus* sequences, thereby enhancing our comprehension of its diversity and ecological function. Metagenomic sequencing has been widely used for studying microbial community across various environments, including oceans, soils, polar regions, and deep-sea trenches [21,22,23]. It is commonly used to assess overall biodiversity and metabolic functions in specific regions. However, this method often overlooks low-abundance, rare species that may play significant roles in biogeochemical cycles. Fluorescence-activated cell sorting (FACS) is an advanced technique based on flow cytometry that enables the detection and sorting of cells from complex microbial samples using fluorescent signals [24,25]. By directly sorting single *Synechococcus* cell from seawater by FACS and subsequently conducting axenic pure cultures, the diversity of cultivable *Synechococcus* in the laboratory can be expanded [26]. Meanwhile, FACS could capture low-abundant species or specific cells group in complex communities through cell sorting [24,27,28]. Isolating *Synechococcus* cells and performing DNA extraction and high-throughput sequencing on them may enhance the recovery of low-abundant species from metagenomes by simplifying the composition of the target microbial community.

However, FACS coupled with high-throughput sequencing for analyzing the community structure and functional genes of *Synechococcus*, remains underexplored. In addition, comparative analysis of *Synechococcus* between the sequencing results of sorted cells and those from in situ samples has not been conducted. To fill this gap, seawater samples were collected from three stations in the coastal waters of Xiamen in summer (June and August) and winter (March and December). FACS was used to isolate *Synechococcus* cells, which were then subjected to DNA extraction and ITS amplicon sequencing, as well as metagenomic sequencing, to analyze the seasonal variations in community structure and functional genes of the *Synechococcus* community. Our previous studies have investigated the genomics of environmental adaptation in marine *Synechococcus* by merging the FACS samples and the in situ samples [29]. However, this paper analyzes the seasonal variations in *Synechococcus* using twelve FACS samples and also adds comparative analysis of three FACS samples and three in situ samples in August. This study reinforced the significance of combining FACS with high-throughput sequencing in studying community variations and functional dynamics and advanced our understanding of coastal *Synechococcus* diversity.

## 2. Material and Methods

### 2.1. Study Area, Sample Collection and Cell Sorting

Xiamen, a subtropical island in China’s southeast, experiences humid summers and dry winters. It is bordered by the Taiwan Strait to the southeast and the Jiulong River to the southwest. The coastal seawater of Xiamen Island exhibits dynamic environments, shaped by freshwater input from Jiulong River, seawater from the South China Sea, and human activities [30,31]. Under the influence of tides, river inputs, and human activities, this complex and dynamic coastal hydrology significantly affects the diversity of *Synechococcus* in the area. However, there has been no investigation of the temporal variations in the *Synechococcus* community in this area.

We sampled seawater at three distinct coastal stations on Xiamen Island (Figure S1). Station S03 (118.02° E, 24.42° N), at the mouth of the Jiulong River, is significantly influenced by the freshwater discharge with high nutrient concentration [30]. Station S07 (118.24° E, 24.49° N) is influenced by the saline water from the South China Sea. Station S12 (118.15° E, 24.59° N) is positioned to the north and close to Tongan Bay. Seawater samples were collected from three stations in March and December 2019, and June and August 2020. Summer samples include those collected in June and August, while winter samples encompass those gathered in December and March. Surface seawater was obtained using sterile bottles and prefiltered through a 20 μm sieve silk. At each site, 2 L of pre-filtered surface seawater were collected, temporarily stored at 4 °C in the dark. After completing sampling at three sites, the samples were immediately transferred to the laboratory for DNA extraction. Meanwhile, a final concentration of 10% *v*/*v* glycerol was added to 2 mL of prefiltered surface seawater as a cryoprotectant, then stored at −80 °C for FACS analysis.

**Figure 1:**
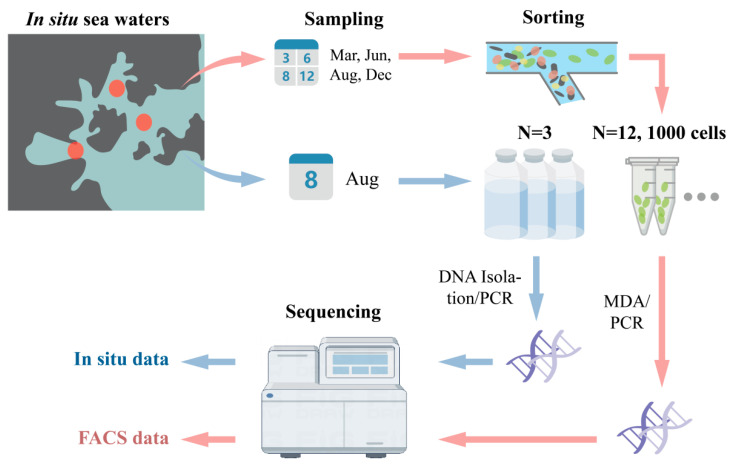
FACS experimental design and high-throughput sequencing workflow. Three in situ samples and twelve FACS samples were collected from three stations during March, June, August and December.

Twelve seawater samples were later thawed, and 1000 *Synechococcus* cells were separately isolated from each sample using a FACS Aria flow cytometer (BD Bioscience, San Jose, CA, USA). The cytometer was equipped with a solid-state laser that provided 13 mW at 488 nm and was fitted with a standard filter setup. Subsequently, the cytometer was configured in purity mode to sort *Synechococcus* cells. The sorting gates were determined based on the population observed in forward scatter (FSC, which serves as a proxy for cell size) and autofluorescence (PerCP, representing chlorophyll autofluorescence) [29]. And, DNA was extracted from the *Synechococcus* cells and sequenced, resulting in twelve FACS data. Meanwhile, DNA was directly extracted from the in situ seawater samples from three stations in August and sequenced to obtain three in situ data. Thus, this study comprises twelve FACS data and three in situ data (Figure 1).

### 2.2. Environmental Parameter Measurements

During the sampling months of March, August, and December, we measured surface seawater temperature, dissolved oxygen, pH, and salinity at three sampling stations using a YSI meter. To address the loss of environmental data in June, surface seawater temperature, dissolved oxygen, pH, and salinity were measured at three existing sampling sites in October. Concurrently, in sampling months (March, June, August, and December), we collected 200 mL of prefiltered surface seawater from each station into sterile bottles for the determination of nitrogen and phosphorus salt concentrations using a PowerMon Kolorimeter AA3 (Bran + Luebbe, Charlotte, NC, USA). Additionally, 1.5 mL of prefiltered surface seawater was transferred into sterile frozen tubes. A final concentration of 1% glutaraldehyde was added, mixed thoroughly, and the samples were stored in liquid nitrogen for subsequent measurement of *Synechococcus* abundance, and measured as previously described [32].

### 2.3. DNA Extraction and Sequencing Data Treatment

DNA was extracted from *Synechococcus* cells isolated by FACS using the Single Cell Kit (Vazyme, Nanjing, China), which is effective for a range of starting cell numbers between 0 and 1000. Meanwhile, in August, 2 L of prefiltered surface seawater from each of the three stations was separately passed through a 0.22 μm polycarbonate membrane. (47 mm, Millipore, Burlington, MA, USA). Polycarbonate membranes, widely used in seawater sample pretreatment and DNA extraction, can effectively retain microbial cells and enhance DNA recovery and purity. The three membranes were then stored at −80 °C for DNA extraction. DNA extraction was conducted on 0.22 μm polycarbonate membranes using the HiPure Soil DNA 96 Kit (Magen, Guangzhou, China). The extracted DNA of twelve FACS samples and three in situ samples was assessed the quality and concentration using a QSEP100 bioanalyzer (BiOptic Inc., New Taipei City, Taiwan) and Qubit 3.0, respectively. Then, DNA libraries were subjected to 2 × 150 bp paired-end sequencing on the Illumina HiSeq platform (Illumina, San Diego, CA, USA). In this study, data preprocessing was carried out as described in previous research [29], where *Synechococcus*-classified sequences (scaffolds ≥ 1 kb) from twelve FACS samples were annotated using the KEGG database to analyze seasonal variations in functional genes. We assessed *Synechococcus* community composition in samples by using the ITS region amplicon sequencing. Amplification of *Synechococcus* ITS sequences was performed using primers 16S-F (TGGATCACCTCCTAACAGGG) and 23S-R (CCTTCATCGCCTCTGTGTGCC) [33]. The amplicons were sequenced on the PacBio platform, generating sequences that were subsequently quality-controlled by Cutadapt (v1.9.1). Unique sequences were identified to determine representative sequences of operational taxonomic units (OTUs) by Vsearch (v1.9.6) [34]. All effective reads were aligned with representative sequences by Qiime (v1.9.1), and those with over 97% similarity were grouped into the same OTU, creating abundance table [35]. This is based on widely accepted practices in microbial ecology.

### 2.4. Community Composition Analysis

Firstly, the Qiime plugin was used to classify all OTUs with the SILVA ribosomal RNA database. To explore *Synechococcus* diversity, OTUs were filtered to retain only those classified within the phylum cyanobacteria. Then, representative sequences of all cyanobacteria OTUs were assigned to cyanobacteria ecotypes by BLASTN against the reference database with >90% identity and >50% coverage. The similarity and coverage thresholds for ecotype classification were stringently set due to the pre-screening of non-cyanobacterial sequences. These thresholds were referenced from relevant literature [36]. The construction of the reference database is derived from multiple literature sources [8,29,36]. In our previous study, a phylogenetic tree has been constructed and two previously undefined clades (XM1 and XM2) were identified, and these two clades were included in the reference database [29].

### 2.5. Statistical Analysis

We used R package ggplot2 for Pearson correlation analysis and significance testing between *Synechococcus* abundance and environmental factors. Alpha diversity of the *Synechococcus* community in the coastal waters of Xiamen was analyzed with Qiime (v1.9.1) through random sequence sampling. Principal coordinate analysis (PCoA) was performed at the OTU level, permutation multivariate analysis of variance (PERMANOVA) was applied to assess differences in community composition across samples, with 999 permutations for significance testing [37]. To evaluate the relationship between clades distribution and environmental variables, Redundancy analysis (RDA) was performed using CANOCO V5.0. Phylogenetic clade proportions were Hellinger-transformed [9], which can effectively mitigate the impact of “0” values in species abundance data on the analysis. Environmental variables were log-transformed to transform variables to approximate normal distribution, reducing the impact of outliers on analysis. The statistical significance of explanatory variables was determined through a Monte Carlo test.

## 3. Results

### 3.1. Environmental Parameters and Synechococcus Abundance Around Xiamen Island

**Figure 2:**
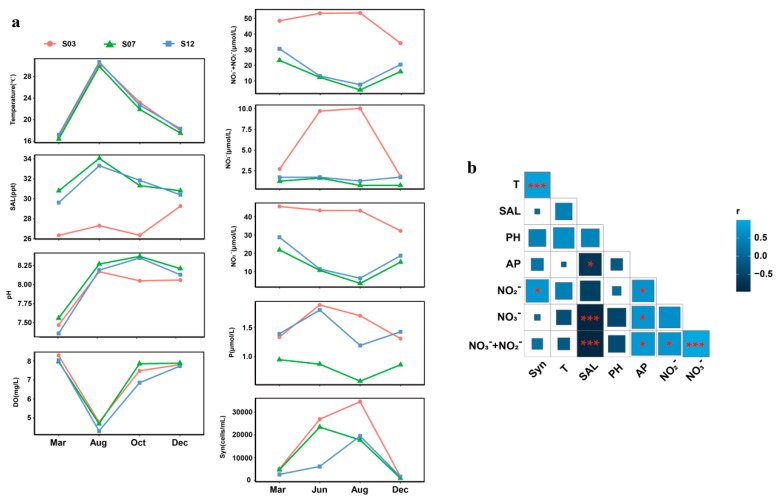
Temperature, Salinity, pH and DO from March to December 2019 and August to October 2020. NO_2_^−^, NO_3_^−^, P and Syn data are presented from March and December 2019, and June and August 2020 (**a**). Syn indicates the abundance of *Synechococcus,* while P indicates the concentration of PO_4_^−^. Pearson correlations between environmental factors and *Synechococcus* abundance (**b**). Square color represents a positive or negative correlation, and square size represents the correlation coefficient. *, *p* < 0.05; ***, *p* < 0.001.

From March 2019 to October 2020, surface seawater samples were collected to measure environmental parameters and *Synechococcus* abundance. Environmental factors showed distinct temporal and spatial patterns (Figure 2a). Water temperature was consistent across all stations. In March, temperatures were slightly lower than in December, reflecting the colder winter marine conditions, whereas June and August exhibited higher temperatures, indicative of the summer season. Seawater temperatures ranged from 16.4 °C to 30.7 °C. Except for a slightly lower pH at station S03 in October, pH levels were similar across stations. Dissolved oxygen levels were consistent, lower in summer and higher in winter. Station S03, influenced by freshwater input, had lower inorganic salt concentrations. In contrast to the typical seasonal trend, S03 showed an unusual increase in inorganic salt concentration in December. Freshwater influx also brought nutrients, leading to higher NO_2_^−^ + NO_3_^−^ and PO_4_^−^ concentrations at S03 compared to other stations.

*Synechococcus* abundance exhibited clear temporal and spatial patterns, with higher levels at S03. Peak abundance occurred in summer, with S03 in August reaching 3.46 × 10^4^ cells/mL. *Synechococcus* abundance decreased in winter, with the lowest at S12 in March, at 2.65 × 10^3^ cells/mL. Pearson correlation analysis was performed between environmental parameters and *Synechococcus* abundance for the months of March, August, and December. A significant positive correlation was detected between temperature and *Synechococcus* abundance (*p* < 0.001), and a positive correlation between NO_2_^−^ concentration and abundance (*p* < 0.05) (Figure 2b). Significant correlations were also observed among environmental variables, notably between nutrient and salt concentrations.

### 3.2. Comparative Analysis of Synechococcus Community Composition

**Figure 3:**
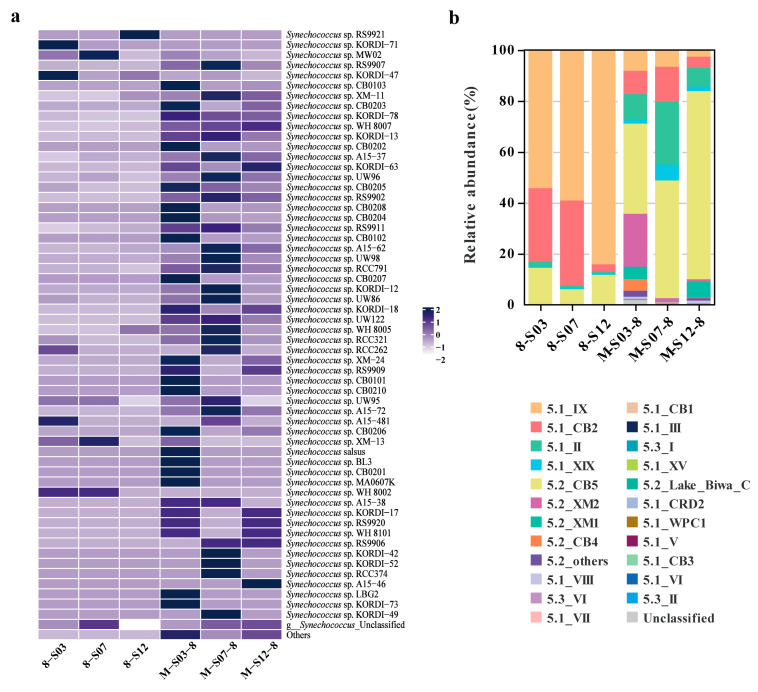
Relative abundance of *Synechococcus* strains (**a**) and clades (**b**) at each station in August. 8-S03, 8-S07, 8-S12 represent the FACS samples; M-S03-8, M-S07-8, and M-S12-8 represent the in situ samples.

A total of 12,777 OTUs (73,710 reads) were detected from 15 samples (three in situ samples and twelve FACS samples) in the coastal waters of Xiamen. Of these, 324 OTUs (2219 reads, 3%) not assigned to the phylum cyanobacteria were discarded. The comparison results of the representative sequences of OTUs with the reference database indicate that 12,348 OTUs (71,342 reads) of cyanobacteria were classified into S5.1, S5.2 and S5.3 of *Synechococcus*, and 105 OTUs (149 reads) were unclassified. In August, 10,251 reads (35%) classified as *Synechococcus* strains, 17,559 reads (60%) assigned to unclassified *Synechococcus* strains, and 1647 reads (5%) not assigned to *Synechococcus* from three in situ samples and three FACS samples. While most *Synechococcus* strains were detected in the three in situ samples with lower or absent abundance in the three FACS samples in August, several strains, such as *Synechococcus* sp. KORDI-71, *Synechococcus* sp. KORDI-47, *Synechococcus* sp. MW02, and *Synechococcus* sp. RS9921, exhibited higher relative abundance in the FACS samples (Figure 3a). Additionally, *Synechococcus* sp. WH 8002 was unique to the FACS samples, and underdetected in the in situ samples.

The in situ samples from three stations identified 22 clades of marine *Synechococcus* in August, whereas FACS samples from the same stations detected only 8 clades (Figure 3b). The core clades detected in the FACS samples were IX, CB2, and CB5, and these core clades were also detected in the in situ samples. In addition, in situ samples detected a high portion of the clade II. The Shannon diversity index detected in the in situ samples varied from 9.57 (S12) to 10.21 (S03), contrasting with the range of 3.52 (S12) to 6.58 (S03) for FACS samples in the coastal waters of Xiamen in August (Figure S2).

### 3.3. Synechococcus Diversity and Their Seasonal Variations

**Figure 4:**
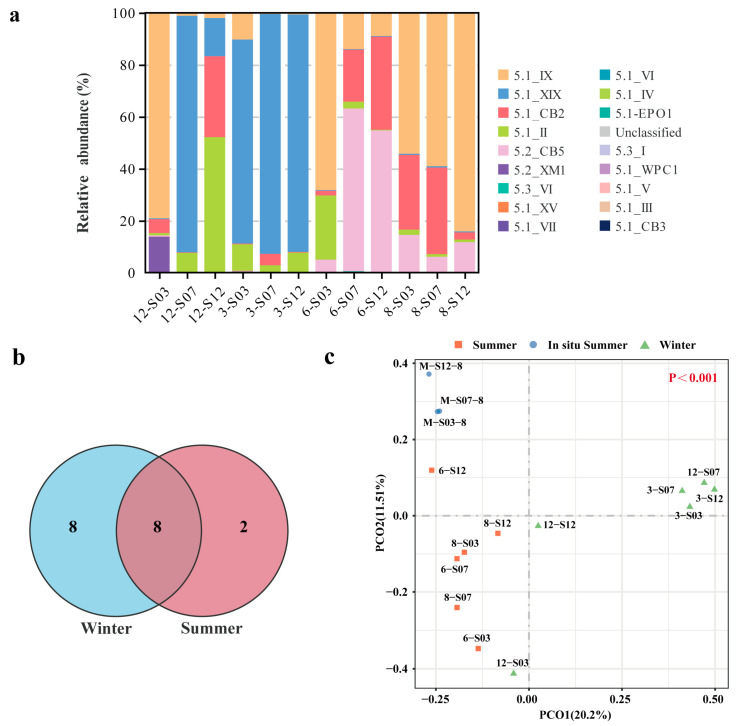
Relative abundance of *Synechococcus* clades at different stations (months) (**a**). Venn diagram of *Synechococcus* clades in winter and summer (**b**). PCoA exhibits the variation in *Synechococcus* community at the operational taxonomic units (OTUs) level across different stations and months (**c**). The term “Summer” represents the FACS samples from June and August, “in situ Summer” represents the in situ samples from August, and “Winter” represents the FACS samples from December and March.

During winter (December and March) and summer (June and August), a total of 17 *Synechococcus* clades from three subclusters (S5.1, S5.2 and S5.3) were identified at three stations by twelve FACS samples. S5.1 and S5.2 were the most prevalent, with core clades including IX, CB2, XIX, II, and CB5 (Figure 4a). S5.1 dominated in winter, with XIX and II as core clades. However, high levels of IX and XM1 were unique to station S03 in December. In summer, S5.1 and S5.2 dominated, IX, CB2 and CB5 were core clades. The coastal waters of Xiamen shared eight *Synechococcus* clades between summer and winter samples, with eight unique clades in winter and three unique clades in summer (Figure 4b).

PCoA revealed that the *Synechococcus* community composition between summer and winter samples exhibited significant differences, with the PCo1 and PCo2 axes collectively explaining 33.7% of the variations in community composition (*p* < 0.001) (Figure 4c). The summer group included FACS samples from three stations in June and August, demonstrating consistent *Synechococcus* composition. Conversely, the winter group comprised FACS samples from the same stations in March and December, also showing similar *Synechococcus* composition, except for the 12-S03 and 12-S12 samples in December, which diverged possibly due to environmental fluctuations. In the coastal waters of Xiamen Island, the Shannon diversity index of the *Synechococcus* community varied between 3.52 and 7.73 during four seasonal sampling months revealed by FACS data, while the ACE richness index spanned from 1600 to 5229 (Figure S3). The highest Shannon diversity was observed at station S03 in March, and the highest ACE richness was recorded at station S12 in December.

### 3.4. Relationship Between Synechococcus Clades and Environmental Factors

**Figure 5:**
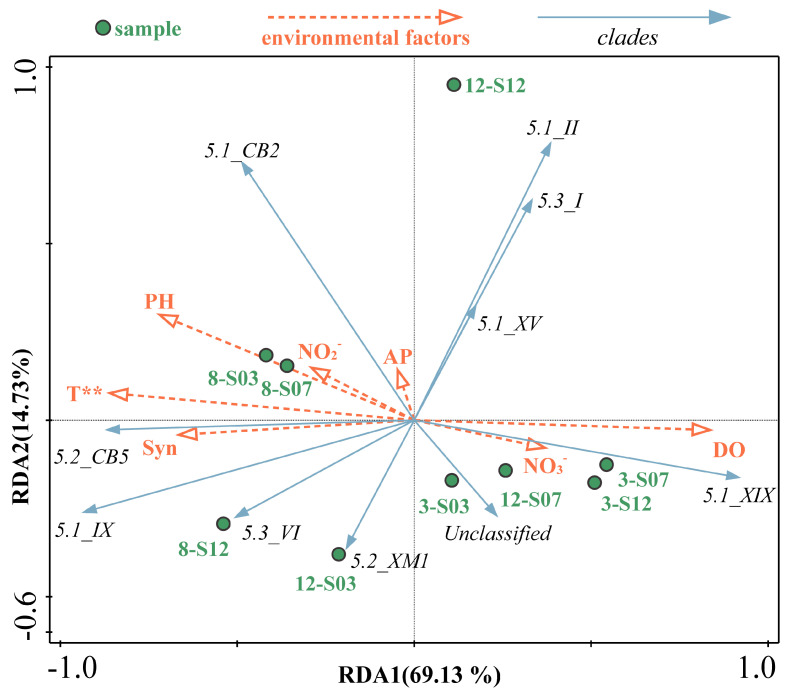
RDA showed the relationship between environmental factors and *Synechococcus* clades. **, Environmental factors and clade changes were significantly correlated (*p* < 0.01).

RDA was used to analyze the relationship between *Synechococcus* clades and environmental factors. The first two axes of RDA accounted for 83.86% of the variations in *Synechococcus* clades composition in the coastal waters of Xiamen (Figure 5). Temperature was identified as the most influential factor, affecting *Synechococcus* community structure (Monte Carlo test; *p* < 0.01) and explaining 52.2% of the variations in clade composition. Clade IX and CB5 were positively correlated with temperature. Winter samples, with the exception of 12-S03, clustered on the right side and were separated from the summer samples.

### 3.5. Synechococcus Metabolic Pathways and Their Seasonal Variations

**Figure 6:**
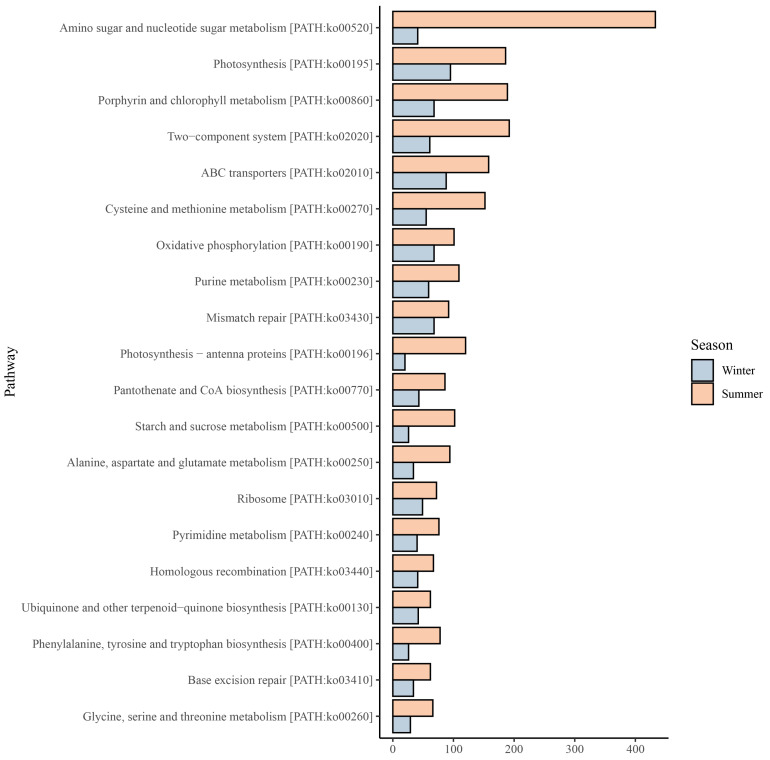
The top 20 metabolic pathways ranked by the number of associated functional genes in winter and summer samples.

Here, we employed the KEGG database to annotate functional genes of twelve FACS samples collected from the coastal waters of Xiamen, and a total of 219 different functional genes were annotated. The results demonstrated that in the summer samples, the number of functional genes associated with amino sugar and nucleotide sugar metabolism was the highest, which was significantly greater than that in the winter samples (Figure 6). In the winter samples, the functional genes related to photosynthesis were the most abundant, but the number was still lower than that in the summer samples. Moreover, across both seasons, genes related to porphyrin and chlorophyll metabolism, two-component systems, and ABC transporters maintained relatively high numbers. However, for the majority of metabolic pathways, the number of relevant functional genes was higher in summer.

## 4. Discussion

### 4.1. Detection of Low-Abundance Synechococcus Strains by Combining FACS with High-Throughput Sequencing

Metagenomics provides a comprehensive approach to uncovering the genomic, metabolic, and phylogenetic diversity of the *Synechococcus* community [38,39]. Yet, metagenomic methods often fail to elucidate lower abundance members of microbial community. Combining cell sorting with metagenomic sequencing may improve the genome recovery of low-abundant species from complex communities [27]. Here, some strains of *Synechococcus* that are rarely detected or absent in the in situ samples were detected in the FACS samples (Figure 3a). These results demonstrate that FACS decreases the complexity of environmental microbial communities through cell sorting, offering a distinct advantage for investigating rare and low-abundance species in marine environments. This finding is consistent with another study of soil, which has shown that targeting sorting and high-throughput sequencing could detect rare species [40]. While FACS and ITS amplicon sequencing may be beneficial for examining low-abundance *Synechococcus*, its limitation lies in sorting only 1000 cells, which leads to a lower sequencing depth. Therefore, the diversity and richness of the *Synechococcus* community in the FACS samples are substantially lower compared to the in situ samples (Figure S2). Meanwhile, *Synechococcus* sp. WH 8002 was uniquely detected in the FACS samples. This may be attributed to the potential biases introduced by FACS, as well as its advantages in isolating specific strains. However, while in situ sequencing may not detect low-abundance strains, FACS data could complement several findings, thereby enhancing our understanding of microbial diversity in marine environments. Consequently, combining FACS with ITS amplicon sequencing may be a valuable tool for analyzing marine microbial diversity.

Core clades IX, CB2, and CB5, detected in the FACS samples, are also present in the in situ samples, but there are discrepancies in their relative proportion (Figure 3b). Additionally, some clades that are abundant in the in situ samples, such as II and XM2, are barely detected in the FACS samples, possibly due to differences in sampling depth. For instance, on the southern California coast, the read ratios of clade I and clade IV in the rpoC1 library were different from those in the ITS amplicon library [36]. Moreover, substantial differences in *Synechococcus* community composition are observed when comparing DNA and cDNA sequences [9].

### 4.2. Seasonal Variations in Synechococcus and Their Relationship with Environmental Factors

Marine *Synechococcus* exhibits high genetic diversity, with over 20 clades identified worldwide. Typically, marine regions host about 6 to 13 clades [41,42]. Estuaries, characterized by complex hydrological conditions, often have a high abundance of *Synechococcus*. For instance, in Hong Kong’s estuary, 17 *Synechococcus* clades were identified through environmental *rpoC1* gene sequencing, along with cyanobacteria and freshwater *Synechococcus* [43]. In this study, twelve FACS samples and three in situ samples identified 24 distinct clades within three sub-clusters (S5.1, S5.2, S5.3) in the coastal waters of Xiamen. This finding highlights the high diversity of *Synechococcus* in this area. The distribution of *Synechococcus* extends from the equator to the poles, and from shore to open oceans. S5.1 is globally widespread, S5.2 is typically detected in estuary and freshwater environment, and S5.3 is more abundant only in the oligo-trophic or warm open oceans [19,44]. Our study aligned with the known distribution patterns of *Synechococcus,* S5.1 was the most prevalent in all samples, S5.2 was abundant only in summer samples, and S5.3 showed consistently low abundance in all samples (Figure 4a). Seasonal and spatial variations in *Synechococcus* community were detected in multiple marine areas [9,45]. Our study also confirmed significant seasonal variations in *Synechococcus* community composition in the coastal waters of Xiamen by combining FACS with ITS amplicon sequencing. II and XIX were core clades in winter waters, IX, CB2 and CB5 were core clades in summer waters (Figure 4a). Clade XIX is a newly identified lineage of *Synechococcus* in 2012, with a limited oceanic distribution [46]. Clade XIX only appears in particular sea areas and confirming if this distribution indicates a specific niche adaptation requires further investigation [19]. However, our results showed that this clade had high abundance during winter in the coastal waters of Xiamen, which might increase our understanding that strains of this clade prefer coastal winter environment.

Environmental changes can affect the physiology and gene expression of *Synechococcus*, potentially driving its adaptive evolution [19]. The phylogenetic diversity of *Synechococcus* represents physiological or ecological diversity, with different evolutionary lineages occupying distinct ecological niches. Consequently, changes in environmental conditions could lead to variations in the community composition of *Synechococcus*. Environmental conditions partly determine the community composition of *Synechococcus*, *Prochlorococcus*, and other small phytoplankton in the study area [47,48]. In the coastal waters of Xiamen, temperature is a significant factor influencing *Synechococcus* community composition (*p* < 0.01, Figure 5), and a temperature-influencing pattern has been observed globally [49]. Additionally, salinity, nutrients, iron availability and chlorophyll content significantly impact the distribution of *Synechococcus* various clades [50,51]. For instance, a shift in dominance from freshwater *Synechococcus* to the combination of phylogenetic subcluster 5.2 and freshwater *Synechococcus* occurs as salinity increases in salt-wedge estuaries [44]. In our study, in December, a notable rise in inorganic salt concentration at station S03 potentially stimulated the growth of the XM1 clade of phylogenetic subcluster 5.2.

The niche partitioning among *Synechococcus* populations in both typical and specialized marine environments is governed by their adaptive mechanisms, which operate through environmental restrictions on the expression and regulation of functional genes and concomitant shifts in ecological functions [19]. Our study revealed that the most enriched metabolic pathways in *Synechococcus* populations from Xiamen coastal waters were amino sugar and nucleotide sugar metabolism, photosynthesis, porphyrin and chlorophyll metabolism, two-component systems and ABC transporters. Notably, summer samples exhibited a significantly higher number of functional genes associated with these pathways compared to winter samples (Figure 6). This may be related to the high temperature and strong sunlight in summer. Temperature directly affects phytoplankton by influencing their growth and metabolic rates [52,53]. In summer, high light intensity and elevated temperature stimulate the expression of functional genes related to photosynthesis and pigment metabolism in *Synechococcus* populations, enabling them to maximize the utilization of light energy for growth and metabolism. The high temperature in summer promotes the growth of *Synechococcus* [54,55] and may accelerate the metabolic rate of cells, resulting in increased expression of functional genes related to energy metabolism and substance synthesis in summer. For example, the high abundance of *Synechococcus* in summer requires substantial amounts of amino sugar and nucleotide sugar to construct new cellular structures. In contrast, the low temperatures in winter may restrict the physiological activities of *Synechococcus* cells, leading to a decrease in the expression of most functional genes. In summer, microbial communities may enhance certain metabolic pathways through interspecies synergies or competition. *Synechococcus* coexists and competes with various microalgae, some metabolites of *Synechococcus*, such as fatty acids, hydrocarbons, phenols, terpenes, and indoles, can act as allelochemicals to inhibit the growth of competing species [19,56]. Our results revealed that the biosynthesis pathways of ubiquinone and other terpenoid-quinones biosynthesis were among the top 20 metabolic pathways in the *Synechococcus* community, and the number of relevant functional genes associated with this pathway was significantly higher in summer. Meanwhile, higher nutrient concentrations in summer seawater may also boost *Synechococcus* community growth and metabolism, and our study detected higher nitrogen and phosphorus concentrations in summer seawater samples, particularly at site S03. In total, *Synechococcus* populations adapt to seasonal environmental changes by regulating the expression of different functional genes to maintain cell growth, metabolism, and survival.

Our research on marine *Synechococcus* diversity and its environmental responses offers insights for broader marine ecosystem studies. Understanding *Synechococcus* diversity and its seasonal and environmentally driven changes can refine models of marine productivity and carbon sinks, which is crucial for the oceanic carbon cycle and other biogeochemical processes. Meanwhile, the seasonal dynamics of *Synechococcus* communities and their metabolic responses to environmental factors like temperature and nutrients can guide future research on ecological adaptation mechanisms in marine microbes. This is essential for predicting the impacts of climate change on marine ecosystems.

## 5. Conclusions

Overall, this study combined FACS with high-throughput sequencing to analyze the seasonal variations in community structure and functional genes of *Synechococcus* in the coastal waters of Xiamen. FACS and ITS amplicon sequencing could detect several low-abundance *Synechococcus* strains underdetected by the in situ samples. FACS data revealed seasonal variations in the *Synechococcus* community composition in these waters concurrently, significantly correlated with temperature. Furthermore, FACS and metagenomic sequencing revealed that the predominant metabolic pathways of *Synechococcus* populations, with *Synechococcus* exhibiting enhanced metabolic activity during summer. In total, FACS combined with high-throughput sequencing offers new insights into microbial community research. This study reveals seasonal *Synechococcus* community changes, enhancing our understanding of how they respond to environmental changes in community composition and metabolic activities. As key marine primary producers, *Synechococcus* community structure and functional variations directly impact marine ecosystem health and are crucial for grasping oceanic carbon cycling and other biogeochemical processes. Future research could expand the sampling scale to analyze the effects of other environmental factors on *Synechococcus* communities. Additionally, the integration of FACS with high-throughput sequencing could be applied to the study of other microbial communities.

## Data Availability

The ITS amplicon sequencing raw data that support the conclusion of this study are deposited in the NCBI database under BioProject ID PRJNA1117049, and metagenome data under BioProject ID PRJNA1030774.

## Supplementary Materials

The following supporting information can be downloaded at: https://www.mdpi.com/article/10.3390/microorganisms13040764/s1, Figure S1: Three sampling stations in the coastal waters of Xiamen Island, China; Figure S2: The Shannon diversity index (a) and ACE index (b) of the *Synechococcus* community of three stations in August; Figure S3: The Shannon diversity index (a) and ACE index (b) of the *Synechococcus* community at different stations (months).
