# Supplementary material for: Seasonal Variations of Community Structure and Functional Genes of Synechococcus in the Subtropical Coastal Waters: Insights from FACS and High-Throughput Sequencing
Source: Microorganisms. 2025 Mar 27;13(4):764. doi: 10.3390/microorganisms13040764 (PMC12029665; doi:10.3390/microorganisms13040764)
Supplement: Supplementary file 1 [file microorganisms-13-00764-s001.zip › microorganisms-3518503-supplementary.pdf]

**Supplement**

# Seasonal Variations of Community Structure and Functional Genes of *Synechococcus* in the Subtropical Coastal Waters: Insights from FACS and High-Throughput Sequencing

Zhenzhen Song[1,2], Ting Zhang[2], Yantao Liang[1,3*], Andrew McMinn[1,4], Min Wang[1,3,5,6], Nianzhi Jiao[2], Tingwei Luo[2*]

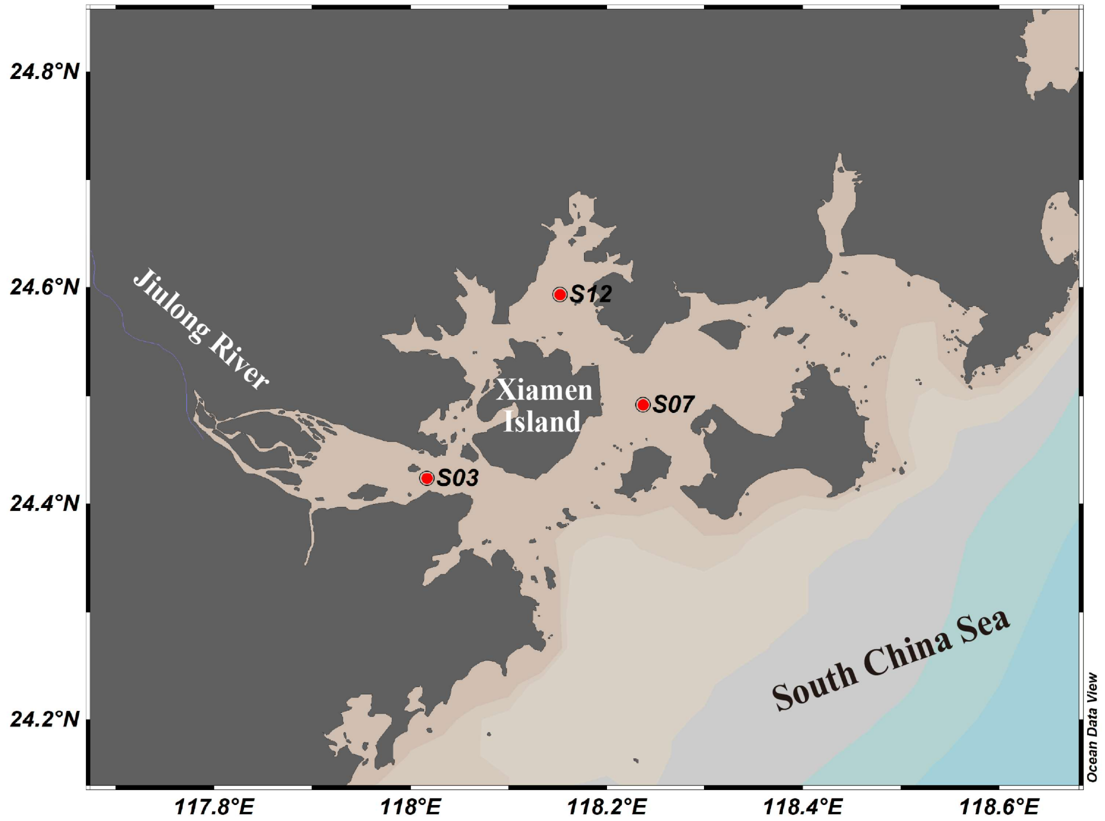


**Figure S1** Three sampling stations in the coastal waters of Xiamen Island, China.

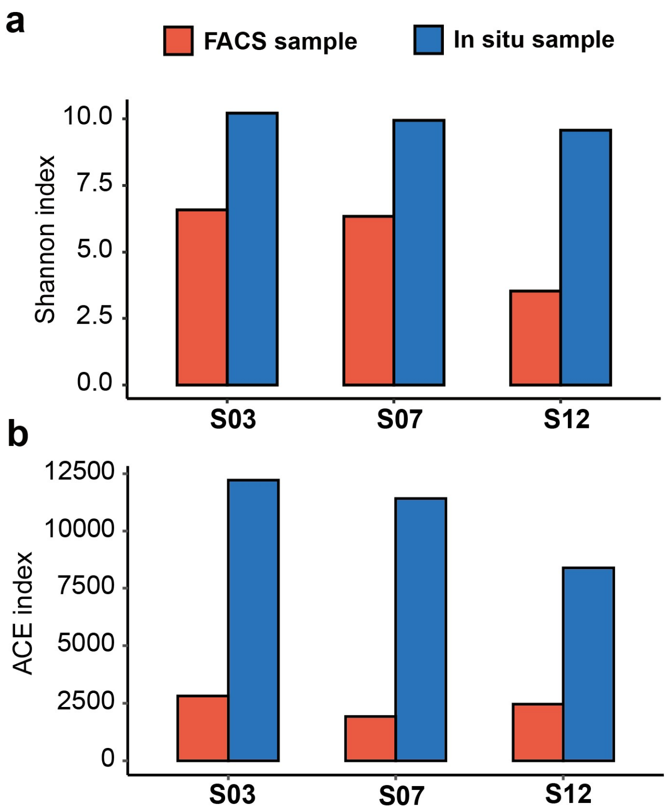


**Figure S2** The Shannon diversity index (a) and ACE index (b) of the *Synechococcus* community of three stations in August.

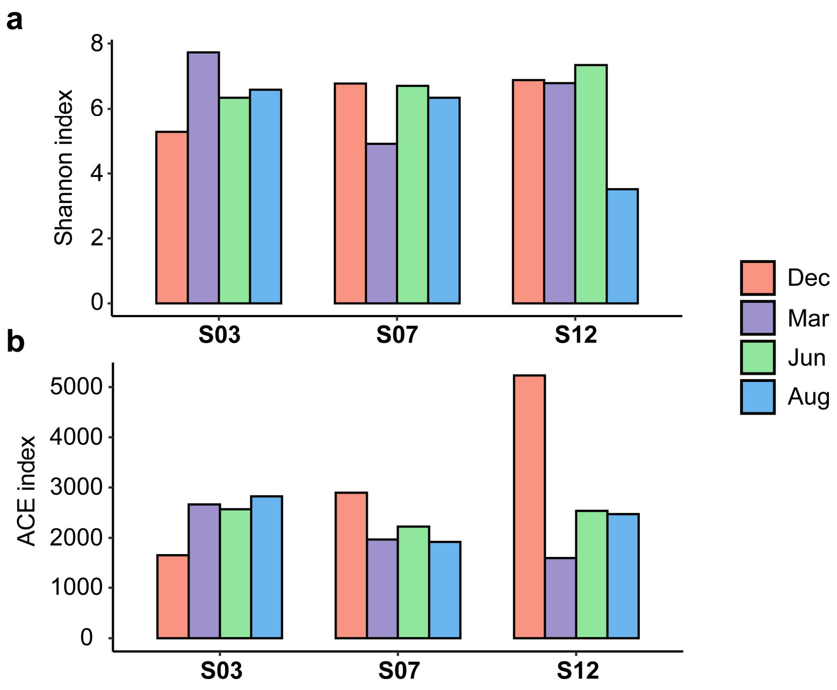


**Figure S3** The Shannon diversity index (a) and ACE index (b) of the *Synechococcus* community at different stations (months).
